# Crystal structure of a closed ternary complex of a HNA Reverse Transcriptase in complex with a HNA/DNA duplex

**DOI:** 10.1371/journal.pone.0351418

**Published:** 2026-07-31

**Authors:** Cédric Gutfreund, Mikhail Abramov, Frédérick Coosemans, Philipp Holliger, Piet Herdewijn, Karin Betz, Andreas Marx

**Affiliations:** 1 Department of Chemistry, University of Konstanz, Konstanz, Germany; 2 Department of Medicinal Chemistry, KU Leuven, Leuven, Belgium; 3 MRC Laboratory of Molecular Biology, Cambridge, United Kingdom; University of the Pacific, UNITED STATES OF AMERICA

## Abstract

1,5-Anhydrohexitol nucleic acid (HNA) is a promising xeno nucleic acid (XNA) for applications such as aptamers and catalysts, due to its favourable physico-chemical properties. Realizing this potential requires efficient and high-fidelity polymerases capable of processing HNA. A key component are HNA reverse transcriptases that convert HNA into DNA, an essential step in standard SELEX workflows. Although HNA reverse transcriptases have been generated by directed evolution, structural insight is essential to guide further enzyme engineering. Here, we report the 2.8 Å crystal structure of the engineered HNA reverse transcriptase KOD-H4, derived from the B-family DNA polymerase of *Thermococcus kodakarensis*, captured in a closed ternary complex with dATP, a 3’-terminated primer and a mixed HNA/DNA template. Compared to a previously reported open ternary KOD-H4 structure, the presented structure adopts a more closed conformation with increased finger and thumb domain closure and formation of a canonical Watson–Crick–Franklin base pair at the insertion site. Direct downstream nucleotides show more distorted base pairing and one HNA residue transits from the unusual ^1^C_4_ conformation it adopted in the open complex to the ^4^C_1_ hexitol sugar conformation. These findings demonstrate that KOD-H4 can form a closed, pre-catalytic complex resembling that of the wildtype enzyme with natural substrates, and reveal state-dependent conformational flexibility of HNA. Such flexibility should be considered in the design and optimization of enzymes that process HNA.

## Introduction

**Fig 1:**
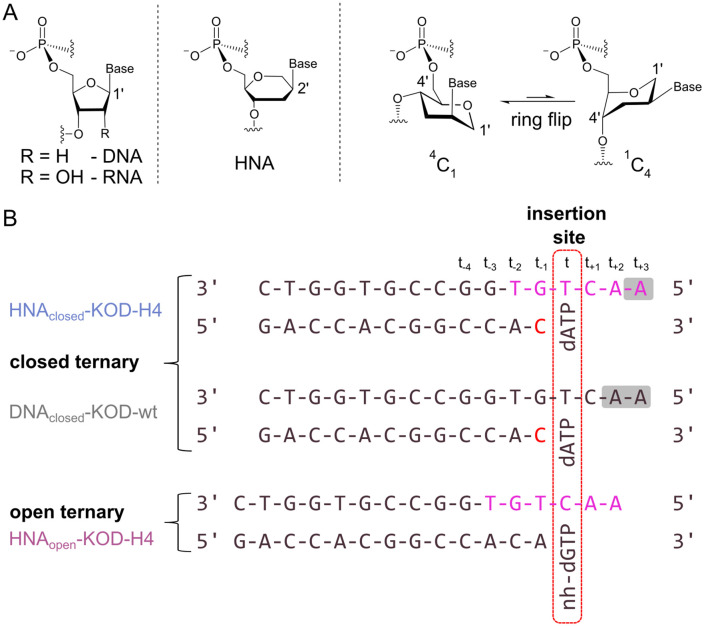
HNA and DNA structure and comparison of the sequences used for crystallization. A) DNA and HNA chemical structure and the two possible chair conformations ^4^C_1_ and ^1^C_4_. B) Templates used for the crystallization of KOD complexes compared in this paper. The red rectangle marks the insertion site. HNA nucleotides are shown in magenta and the ddC nucleotides in red. Unmodeled nucleotides are underlaid with gray. HNA_closed_-KOD-H4 (PDB:9R83), DNA_closed_-KOD-wt (PDB:5OMF) and HNA_open_-KOD-H4 (PDB:9EMI) are shown in this order from top to bottom with either natural dATP or a non-hydrolyzable dGTP analogue (nh-dGTP) bound in the active site.

Xeno nucleic acids (XNAs) are a class of promising nucleic acid analogues, featuring modified sugar moieties that exhibit a variety of intriguing properties, including enhanced thermal, chemical, and enzymatic stability [1–9]. These characteristics render them ideal candidates for utilization in molecular medicine, for example as aptamers or therapeutic oligonucleotides, or for potential long-term data storage [1–10]. One promising XNA candidate is 1,5-Anhydrohexitol nucleic acid (HNA) (Fig 1A), which is composed of a hexitol sugar, as opposed to the naturally occurring ribofurano-pentose in natural nucleic acids (Fig 1A). Using HNA oligonucleotides it was for example possible to select high-affinity aptamers against rat vascular endothelial growth factor 164 from a library of MeORNA-HNA sequences [7]. This was achieved using an evolved DNA-dependent HNA polymerase to generate the HNA library from a randomized DNA library and an XNA-dependent DNA polymerase (or reverse transcriptase) to convert the selected XNA sequences to DNA. These aptamers exhibited the desired property of exceptionally high stability towards nucleases in human serum.

Before DNA polymerases that are able to process ribose-modified substrates were found, these unnatural systems had to be chemically synthesized, which limited their potential applications. The development of XNA-processing enzymes mainly relies on thermostable DNA polymerases from either the A- or B-family, such as the DNA polymerase of *Thermus aquaticus* (Taq) or *Thermococcus gorgonarius* (Tgo). Evolution of these enzymes are complemented by a few structural studies that demonstrate the acceptance of the modified substrates on a molecular level. In the field of HNA, Samson and co-workers were, e.g., able to crystallize a HNA polymerase (synthesizing HNA from DNA) derived from Tgo (TgoT_6G12) in complex with two hexitol nucleotides in the primer [11]. More recently, our group solved several crystal structures of a HNA reverse transcriptase (synthesizing DNA from HNA) in complex with up to six HNA nucleotides [12]. Faithful XNA reverse transcriptases are essential for advancing biotechnological applications such as optimizing ligands/aptamers [3,7,8,13–16] and catalysts/XNAzymes [5,17–19] by methods like SELEX and directed evolution [11,20–25]. Therefore (in our previous study), we examined a thermostable HNA reverse transcriptase variant of the B-family DNA polymerase *Thermococcus kodakarensis* (KOD-H4), that was based on the Tgo-H4 variant developed in the Holliger laboratory via directed evolution [12,26]. The transfer of the mutations from Tgo to KOD (which have 93% sequence identity) was conducted for the purpose of crystallization. The generated KOD-H4 enzyme includes the following mutations: V93Q, I114T, D141A, E143A, S383K, K429G, F445L, A485L, Y493V, Y496H, Y497M, Y499F, A500E, R501N, I521L, E664K, K726R and N735K. It was demonstrated that the KOD-H4 mutant is proficient in reverse transcription of HNA into DNA [12]. Furthermore, the enzyme was crystallized in a ternary complex with a partially open finger domain (HNA_open_-KOD-H4), which comprises a primer/template (p/t) complex with six HNA modifications in the template and a bound non-hydrolyzable 2’-deoxy-guanosine-triphosphate (nh-dGTP). The structural adaptation of the enzyme to the bulkier HNA moieties was demonstrated by uncovering an unusual chair conformation of the HNA six-membered ring. More precisely, the six-membered ring flips from the more stable canonical ^4^C_1_ conformation (in which the nucleobase is positioned axially) to the less stable ^1^C_4_ conformation (with an equatorial nucleobase position) under certain conditions (Fig 1A). This flip is seen in all three HNA nucleotides (t_-1_ to t_-3_) downstream of the templating nucleotide (t). The flipped ^1^C_4_ conformation, in which the nucleobase is positioned equatorially, mimics the C3’-exo conformation found in natural DNA and RNA. The B-form helix, characterized by C3’-exo and C2’-endo ribose conformations, is the preferred structure of a DNA duplex. [27,28]. On the contrary, the presence of the additional hydroxy group in ribose strongly favors the C3’-endo and C2’-exo pucker and results in A-form helix packing [28]. Therefore, the crystal structure HNA_open_-KOD-H4 revealed that three HNA nucleotides exhibit a conformation that mimics B-form helices. This is in contrast to the published free HNA duplex crystal structures where the duplex closely resembles A-form helices [14,29–31]. The polymerase-induced flip of nucleotide conformation (^4^C_1_⇌ ^1^C_4_) shifts the helical geometry toward a more B-form-like structure within the enzyme’s active site. This helix conformation has been shown to be preferred by B-family polymerases like KOD [32–36]. However, it was not clear if these non-canonical conformations would persist in the closed, catalytically competent conformation of the enzyme.

Here, we report the crystal structure of KOD-H4 with a significantly more closed finger domain (HNA_closed_-KOD-H4,) in a ternary complex. This complex contains the same template including six HNA nucleotides as was used for the previously published open ternary structure (HNA_open_-KOD-H4, PDB:9EMI [12], Fig 1B). In previous attempts to crystallize KOD-H4 in a closed ternary complex, we were unable to obtain structures in which the finger domain of the polymerase was completely closed, as it is observed in the KOD wildtype (wt) (DNA_closed_-KOD-wt, Fig 1B, PDB:5OMF [35]) structure. In contrast to previous setups, where a natural DNA primer and a non-hydrolyzable triphosphate substrate were used, we now used a dideoxy nucleotide terminated primer and a natural dATP as substrate. Crystals were obtained in a different crystallographic space group with two polymerase complexes within the asymmetric unit. The structure presented here provides a further building block for a better understanding of the processing of HNA by DNA polymerases with HNA reverse transcription function. These insights should advance the further development of HNA-processing enzymes with regard to increased specificity and processivity.

## Materials and methods

Kod-H4 was expressed and purified as described by Gutfreund *et. al.* [12]. Figures depicting crystal structures were generated using PyMOL [37]. Root mean square deviation (RMSD) values were determined by superimposing either the entire structures (excluding solvent molecules and ions) or substructures in PyMOL [37] using the default align function after outlier rejection. Omit maps were generated with Phenix [38–40] and are displayed as mesh at 3 σ. To compare the conformations of the HNA nucleotides, the nucleic acid duplexes of the crystal structures were aligned based on the triphosphates using PyMOL [37] and the omit maps were then superimposed using the “matrix_copy” command.

### Oligonucleotides

DNA oligonucleotides were purchased HPLC-purified from Biomers. The oligonucleotides were used without further purification for the crystallization experiments. HNA phosphoramidite synthesis was conducted according to the literature [41]. The oligonucleotides were then assembled using the phosphoramidite approach, purified and verified by mass spectrometry as described previously [12].

### Crystallization

The crystallization procedure was adapted from Gutfreund *et. al*. using sitting drop vapor diffusion [12]. A 12mer DNA primer 5’-d(GAC CAC GGC CA**ddC**)-3’- bearing a final 2’-,3’-dideoxy cytidine modification (ddC) was annealed in a ratio of 1:1 with HNA_3+3_ -Template (5’-d(A*A*C* T*G*T* GGC CGT GGT C)-3’, where the asterisks indicate HNA nucleotides). Then, a 1.2-molar excess of the p/t duplex (with respect to the enzyme) was added to the RT-low buffer (20 mM Tris–HCl, pH 7.4, 50 mM NaCl, 10% (w/ v) glycerol), 20 mM CaCl_2_, together with Kod-H4 (final concentrations of 3.5 mg/ ml) and a 10-fold molar excess of dATP (JenaBioscience). The solution was incubated for 30 minutes at 30 °C, then kept on ice and filtered using a 0.1 μm sterile filter (Ultrafree Centrifugal Filters, Millipore). Crystallization experiments were conducted using a Gryphon robot (Art Robbins Instruments, ARI) and a Morpheus crystallization screen (Molecular Dimensions) in three-well sitting-drop plates (ARI) with final drop volumes of 0.8–0.9 µL. The protein solution was mixed with the reservoir solutions at ratios of 2:1, 1:1, or 1:2 (v/v). The crystals were either frozen directly or cryoprotected using a mixture of ethylene glycol or glycerol (20% v/v) and the respective crystallization condition and stored in liquid nitrogen prior to shipping. The crystals grew in the F10 condition of the Morpheus I screen, which contains 10% w/v PEG 8000, 20% v/v ethylene glycol, and 0.02 M each of D-Glucose, D-Mannose, D-Galactose, L-fucose, D-Xylose, and N-acetyl-D-glucosamine, as well as 0.1 M bicine/trizma base at pH 8.5. KOD-H4 crystallized in space group P2_1_2_1_2_1_ with two enzyme/substrate complexes in the asymmetric unit.

### Data collection & processing

The synchrotron data were collected at beamline P14 operated by EMBL Hamburg at the PETRA III storage ring (DESY, Hamburg, Germany). Data were processed using the XDS package and the XDSGUI graphical interface (https://strucbio.biologie.uni-konstanz.de/xdswiki/index.php/XDSGUI) [42,43]. The high-resolution cutoff was selected based on inspection of electron density maps and refinement behaviour. R-free flags were created using the UNIQUEIFY script [44]. The structure was solved by molecular replacement against a published KOD-H4 structure (PDB: 9EMI [12]). Structure refinement was performed using phenix.refine (using TLS, optimization of ADP-weights and X-ray-weights and NCS) [38–40]. Data quality was assessed using phenix.xtriage [45] which indicated pseudo-translational symmetry of the data for the two complexes in the asymmetric unit. Several surface-exposed regions, particularly helices of the thumb domain, displayed weak electron density and refined to comparatively high B-factors but were retained in the final model. Only residues 749–774 at the C-terminus of each protein chain were omitted due to insufficient electron density. In addition, electron density for the 5’-terminal HNA nucleotide (t_+3_) was weak and it was therefore not modeled in both complexes.

Model building was done in Coot [46,47] and model quality was evaluated by the MolProbity (http://molprobity.biochem.duke.edu) and the PDB validation server (https://validate-rcsb-1.wwpdb.org) [48]. For the refinement of HNA nucleotides, the restraints (cif) files were modified as described by Gutfreund *et. al.* [12]. The crystallographic data tables were generated using phenix.table_one using the default settings with the final model, unmerged data (XDS_ASCII.HKL) and restraints files, respectively [38–40].The final structure has been uploaded to the PDB (PDB: 9R83) and the crystallographic data table is available in the supporting information (S1 Table).

## Results

In our previous study we crystallized two ternary complexes of KOD-H4: one with a fully natural DNA p/t complex (DNA_open_-KOD-H4, PDB:8S84 [12]) and another containing six HNA modifications in the template (HNA_open_-KOD-H4, PDB:9EMI). To set up crystallization, we used a DNA or DNA/HNA-mixed template, an unmodified DNA primer and a non-hydrolyzable dGTP substrate to trap the complex in a ternary state. Although both complexes clearly showed a bound substrate triphosphate, they were not fully closed with respect to their finger domains when compared to the wild-type KOD ternary structure (DNA_closed_-KOD-wt, PDB:5OMF [35]). In addition, the Watson-Crick-Franklin base pairing (WCF-bp) of the templating nucleotide with the incoming triphosphate was rather tilted in both KOD-H4 complexes and the distance between the primer terminus and the alpha-phosphorus of the triphosphate was too large for a direct nucleophilic attack. We concluded that – before catalysis – either a further closing of the finger domain needed to take place to push the triphosphate towards the primer or the primer itself would need to be shifted closer to the substrate triphosphate. Since we could not assess, whether the open ternary state was preferentially trapped due to the KOD-H4 mutations, or whether either the crystallization strategy or crystal contacts played a role, we tried a different strategy with the aim to obtain a fully closed active KOD-H4 complex.

In this study we used a chemically synthesized 2’-3’-dideoxy-cytidine terminated primer and dATP as substrate (Fig 1B). This was necessary as KOD-H4 was shown to be unable to catalyze ddCMP insertion itself in contrast to the KOD-wt enzyme. As a consequence, the resulting structure exhibits only two instead of three HNA nucleotides in the duplexed region (Fig 1B). Crystallization attempts with natural dATP, the ddC primer and catalytically active metal ions (Mg² ⁺ /Mn²⁺) were of limited success, yielding crystals in two different space groups (P2_1_2_1_2_1_ and P2_1_2_1_2), albeit at low resolution (data not shown). Upon closer examination, we found weak electron density for probably one or two additional nucleic acid residues at the 3’-end of the template strand. We speculate that this might stem from a terminal transferase activity of KOD-H4 incorporating nucleotides at the blunt-end side of a p/t duplex [12]. To inhibit this reaction, we switched from using Mg^2+^/Mn^2+^ to catalytically inactive Ca^2+^ ions. This allowed us to crystallize our final KOD-H4 structure with 2.8 Å resolution in space group 19 (P2_1_2_1_2_1_). The asymmetric unit contains two DNA polymerase complexes (A and B) in a closed ternary state, related by translational non-crystallographic symmetry (tNCS). The two complexes are highly similar in conformation, as indicated by an RMSD value of 0.602 (5185 atoms aligned). The two unpaired HNA nucleotides at the free 5’-end of the template prior to the insertion site have poor electron densities and high B-factors and were excluded from the comparison to the previously published open ternary complex. The other three HNA nucleotides are well resolved in both complexes. At the resolution of the data, hexitol sugar conformations slightly deviating from idealized ^1^C_4_ and ^4^C_1_ chair geometries remain compatible with the electron density; therefore, the predominant chair-like conformation was modeled.

Despite the elevated overall B-factors, the active-site region is well resolved, with substantially lower local B-factors observed for residues surrounding the catalytic center. However, as complex B displays lower atomic B-factors than complex A for the protein chain as well as the p/t duplex and the triphosphate substrate (see S2 Table) we conducted further analysis and comparison solely with complex B (hereafter referred to as HNA_closed_-KOD-H4 for clarity). An RMSD comparison of the different ternary complexes clearly shows the increased similarity between the new closed KOD-H4 complex and the closed KOD-wt complex (DNA_closed_-KOD-wt, PDB:5OMF RMSD: 0.661, 5311 atoms aligned). In contrast, the previously published open KOD-H4 ternary complex (HNA_open_-KOD-H4, PDB:9EMI) shows high RMSD values when compared to either the closed KOD-H4 (RMSD: 1.785) or the closed KOD-wt (RMSD: 1.738).

**Fig 2:**
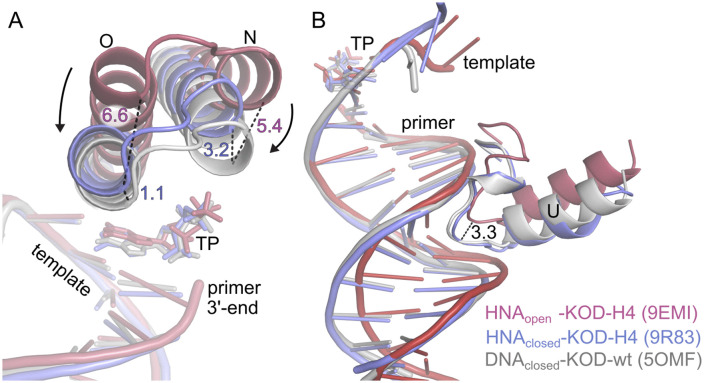
Overlay of both open and closed KOD-H4 HNA structures with the closed KOD-wt structure. A) relative positioning of the finger domain helices (O & N) and the nucleic acid duplex with the bound dATP (in HNA_closed_-KOD-H4 (PDB:9R83) and DNA_closed_-KOD-wt (PDB:5OMF) or nh-dGTP (in HNA_open_-KOD-H4 (PDB:9EMI)); TP = triphosphate. B) thumb domain position in relation to the p/t duplex in the three enzyme complexes. Distances are measured from the Cα of P473 in the O helix, Cα of K468 in the N helix and Cα of A475 in the U helix and are given in Å.

The major differences in the three structures stem from the positioning of the finger and thumb domains as is depicted in Fig 2. HNA_closed_-KOD-H4 clearly exhibits an increased closure of the finger domain compared to the open structure. Here, the O helix of the finger domain is somewhat more alike to the closed wt structure than the N helix (1.1 and 3.2 Å distance, respectively). For comparison, the distances in the previously published open structure compared to the closed wt structure are 6.6 and 5.4 Å for O and N helix, respectively (Fig 2A).

Usually the closed finger domain interacts tightly with the triphosphate in the active site. Here, the direct interactions of polar residues (N491, K487, R460) between the finger domain and the incoming dATP in HNA_closed_-KOD-H4 are similar to DNA_closed_-KOD-wt (S1 Fig). The main difference is the lack of water-mediated interactions of the triphosphate moiety with the side chains of K464 and Q483. These water molecules are not resolved in HNA_closed_-KOD-H4.

The thumb domain positions in the closed wt DNA and KOD-H4 HNA structures are similar, exhibiting the same 3.3 Å displacement compared to the open KOD-H4 HNA structure. Thereby, the flexible loop adjacent to the U helix approaches the nucleic acid duplex more closely than in the open ternary structure (Fig 2B).

**Fig 3:**
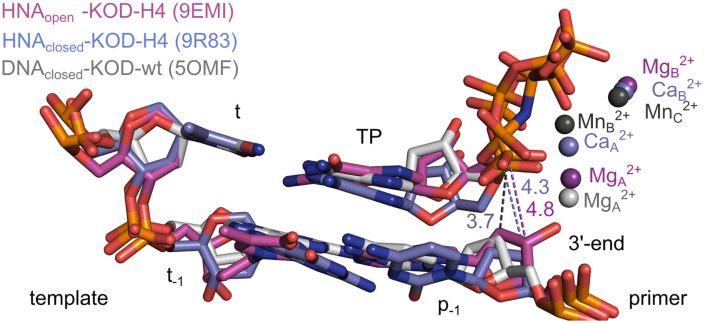
Superimposition of the templating nucleotide, the incoming triphosphate (TP), the first duplexed base pair post insertion site and triphosphate-bound metal ions for HNA_closed_-KOD-H4 (PDB:9R83), HNA_open_-KOD-H4 (PDB:9EMI) and DNA_closed_-KOD-wt (PDB:5OMF). Distances between the 3’-carbon and the α-phosphorus are given in Å.

Along with the closure of the finger domain, the triphosphate substrate also moves closer to the primer 3’-end in HNA_closed_-KOD-H4 (S1 Fig). Whereas in the open structure, the triphosphate moiety of the nh-dGTP adopts a more extended conformation when it binds near the charged residues of the O helix, the substrate dATP in HNA_closed_-KOD-H4 adopts a more curved conformation just as in the closed DNA-bound KOD-wt complex. Comparing closed complexes HNA_closed_-KOD-H4 and DNA_closed_-KOD-wt, the dATP substrates adopt a nearly identical position (Fig S1E and Fig 3; note that in Fig 3 the depicted residues were superposed based on the nucleobase of the templating nucleotide to depict local similarities and differences whereas in S1 Fig the entire complexes were superimposed to show overall differences)

When comparing the distance between the 3′ carbon atom of the terminal primer nucleotide and the α-phosphorus atom of all three complexes depicted in Fig 3, DNA_closed_-KOD-wt exhibits the shortest distance (3.7 Å). The observed distance in HNA_closed_-KOD-H4 (4.3 Å) falls between the longer distance in the open complex of HNA_open_-KOD-H4 (4.8 Å) and the closed complex of the wt (Fig 3). This suggests that the two components would still likely have to be brought a little closer together for an incorporation reaction to proceed. Here, the different ion radii of the Ca² ⁺ ions (114 pm), which were employed instead of the catalytically active Mg²⁺ (86 pm) and Mn²⁺ (97 pm) ions, might contribute to the fact that the triphosphate substrate is not as closely positioned with respect to the primer terminus.

HNA_closed_-KOD-H4, like the HNA_open_-KOD-H4 structure, has two bound metal ions in the enzyme’s active site, unlike the three ions bound in DNA_closed_-KOD-wt (Fig 3).The two Ca² ⁺ ions are bound close to the phosphate chain of the incoming dATP, likely stabilizing the triphosphate by counteracting the negatively charged phosphate groups. Compared to the wt and open KOD-H4 structures, HNA_closed_-KOD-H4 lacks a bridging metal ion between the α-phosphate of the triphosphate and the 3’-end of the primer strand. This is likely due to the use of catalytically inactive metal ions and not a result of the ddCMP-capped primer because the DNA_closed_-KOD-wt retains binding of an additional metal ion.

**Fig 4:**
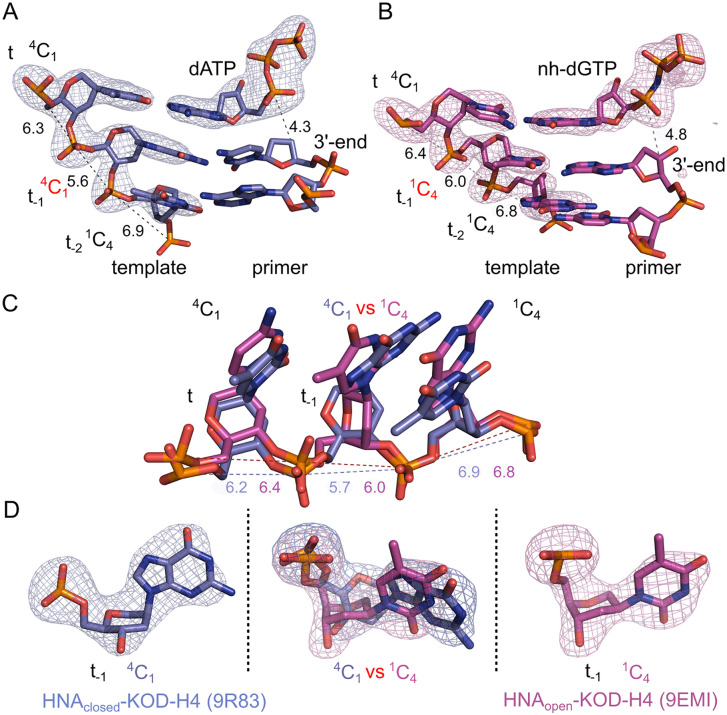
Comparison of the nucleic acid duplex and the incoming triphosphate of HNA_closed_-KOD-H4 (PDB:9R83) and HNA_open_-KOD-H4 (PDB:9EMI). A) Insertion site and the subsequent two base pairs and the incoming dATP in HNA_closed_-KOD-H4. B) Insertion site and the subsequent two base pairs and the incoming nh-dGTP in HNA_open_-KOD-H4. C) Overlay of the HNA nucleotides with inter-phosphate distances at positions t to t_-2_. D) Comparison of the two different nucleotide conformations at position t_-1_ with the respective omit maps (left and right) and their overlay of HNA_closed_-KOD-H4 and HNA_open_-KOD-H4 (middle). Nucleotides are numbered according to the templating nucleotide t, and the chair conformations of the HNA nucleotides are assigned. Omit maps for the HNA and triphosphate residues are shown at 3 σ.

In the previously published HNA structure of the open ternary complex, the incoming nh-dGTP and the templating nucleotide (t) were not oriented in a coplanar fashion. The novel complex exhibits a more coplanar orientation of the incoming natural dATP and the templating nucleotide. These differences are visualized in Fig 4A&B. The orientation of the templating nucleotide’s base moiety and the incoming triphosphate is nearly identical to the wt structure, although the size of the hexitol sugar differs greatly from 2’-deoxyribose in natural DNA (Fig 3 & S2A Fig). This is significant because the HNA templating nucleotides in both the open and closed HNA structures align precisely upon superimposition of the p/t duplex alone, and only the repositioning of the triphosphate establishes proper coplanarity in the case of HNA_closed_-KOD-H4 (Fig 3 & S2B Fig). The repositioning of the incoming triphosphate reduces the distance of the alpha phosphate to the 3’-end of the primer by 0.5 Å. As can be observed in Fig 4, this repositioning has significant effects on the first two base pairs (t_-1_ and t_-2_) of the p/t duplex downstream of the insertion site.

The geometry of the first base pair in the duplex (position t_-1_) differs significantly from the coplanar alignment observed in HNA_open_-KOD-H4 with the nucleobases showing altered buckle and propeller twist (S2C Fig; base pair parameters determined by 3DNA [49] are given in S3 Table). The deviation from coplanarity is also noticeable to a slightly lesser degree in the second base pair post insertion site (Fig 4A, S2C Fig, and S3 Table). A certain level of non-planarity in base pairing is not uncommon and is, e.g., also observed in the DNA_closed_-KOD-wt structure at the t_-1_ position for the dG-ddC pair (S2D Fig).

The most striking change in the p/t duplex is that the HNA hexitol conformation at the t_-1_ position differs from its conformation in the open complex. More precisely, the HNA nucleotide is in the canonical ^4^C_1_ conformation, as opposed to the flipped (^1^C_4_) conformation observed in HNA_open_-KOD-H4 (Fig 4C&D). This finding is consistent with a decrease in inter-phosphate distances from 6.0 Å to 5.7 Å (Fig 4, S4 Table). The overlaid HNA nucleotides of the two KOD-H4 HNA structures are depicted in Fig 4C, where the flipped conformation of the first duplexed nucleotide is indicated. This is evident when comparing the omit maps of the individual residues, which also show the flipped conformation when aligned against each other (Fig 4D).

This change in conformation in response to the closing of the finger domain and repositioning of the triphosphate might be part of the HNA reverse transcription mechanism by KOD-H4. It could indicate that the adaptation to the bulkier HNA residues within the active site of the polymerase is a dynamic process characterized by conformational changes of the nucleic acid duplex throughout the reaction. More precisely we assume that the conformations of the individual HNA nucleotides can change in different reaction states (e.g., binary, ternary open, ternary closed, post insertion) in the way that is most suitable for the enzyme. These additional conformational changes that have to be overcome for the 1,5-Anhydrohexitol compared to the ribose nucleotide (and the likely associated energetic costs) may explain the reduced efficiency of processing of HNA (compared to DNA) by the enzyme. Ultimately, the six-membered ring probably adopts the conformation that is most favorable for the enzyme based on interactions of the p/t complex with the enzyme, on base pairing, base stacking and steric factors.

## Conclusion

We set out to further investigate the HNA reverse transcription mechanism of KOD-H4 by crystallizing it with a natural dNTP rather than a non-hydrolyzable triphosphate analog. Using Ca²⁺ and natural dATP together with a ddCMP-terminated DNA primer, we solved the crystal structure of KOD-H4 in a closed ternary complex with a resolution of 2.8 Å. The closed HNA-KOD-H4 complex exhibits more completely closed finger and thumb domains compared to the previously published open structure. This is accompanied by the formation of a more coplanar WCF-bp interaction between the incoming triphosphate and the templating HNA nucleotide. Therefore, we demonstrate that a closed complex can be formed even in the presence of HNA as a template, which has not been observed previously. As a consequence of the closed complex or as a prerequisite of the enzyme to be able to close, the two base pairs further downstream (at position t_-1_ and t_-2_) show a less coplanar base pairing. While in the open ternary structure both t_-1_ and t_-2_ HNA nucleotides showed an uncommon ^1^C_4_ conformation, in the closed state, t_-1_ now adopts the canonical ^4^C_1_ chair conformation.

These findings provide insights into the HNA reverse transcription of KOD-H4 by highlighting the increased conformational dynamics of HNA, which is essential for accommodating the bulkier sugar moieties within the active site of the enzyme while ensuring efficient processing. Finally, it must be considered that a fully modified HNA/DNA complex may require an opening of the thumb domain since the HNA/DNA duplex may revert to the proposed A-form geometry when located downstream of the active site, as Samson and colleagues previously indicated [11]. These findings highlight the necessary considerations for future polymerase engineering, especially when modeling polymerase-XNA interactions, by emphasizing the need to include a wider range of flexibility for conformational adaptations.

## Supporting information

S1 Fig Comparison of HNA_closed_-KOD-H4 (PDB:9R83) and HNA_open_-KOD-H4 (PDB:9EMI) (A-C) as well as HNA_closed_-KOD-H4 and DNA_closed_-KOD-wt (PDB:5OMF) (A, D & E). Depicted are the finger domain O- and N-helices and selected insertion site residues. Magnesium ions, Calcium ions and Manganese ions bound to the triphosphates are shown as spheres in the colour of the respective structure. Selected water molecules are shown as red spheres. The lower panel shows the templating HNA or DNA nucleotides and the respective bound triphosphate with WCF hydrogen bonding indicated as dashed lines. In the overlays, the water molecules and dashed lines are not shown.
(TIF)

S2 Fig Comparison of the WCF-bp and co-planarity of HNA_closed_-KOD-H4 (PDB:9R83), HNA_open_-KOD-H4 (PDB:9EMI) and DNA_closed_-KOD-wt (PDB:5OMF). A) Comparison of the templating nucleotide and incoming dATP of HNA_closed_-KOD-H4 and DNA_closed_-KOD-wt. B) Comparison of the templating nucleotide and incoming dATP of HNA_closed_-KOD-H4 and nh-dGTP of HNA_open_-KOD-H4. C, D and E) Comparison of the t_-1_ to t_-2_ positions of HNA_closed_-KOD-H4, HNA_open_-KOD-H4 and DNA_closed_-KOD-wt. Conformations of the ribose and hexitol rings are indicated.
(TIF)

S1 Table Data collection and refinement statistics for the HNA_closed_-KOD-H4 (PDB:9R83) structure. Statistics for the highest-resolution shell are shown in parentheses.
(DOCX)

S2 Table Atomic B-factor comparison of the two polymerase complexes (A&B) within the asymmetric unit of HNA_closed_-KOD-H4 (PDB:9R83). (DOCX)

S3 Table Local base parameters as given by 3DNA [49] for the active sites (t to t_-4_) within the duplexed structures of HNA_open_-KOD-H4 (PDB:9EMI), HNA_closed_-KOD-H4 (PDB:9R83) and DNA_closed_-KOD-wt (PDB:5OMF). (DOCX)

S4 Table Inter-phosphate distances of template strands at positions t to t_-3_. HNA nucleotides are marked in magenta and distances were measured with Pymol [37].
(DOCX)
