# Supplementary material for: Crystal structure of a closed ternary complex of a HNA Reverse Transcriptase in complex with a HNA/DNA duplex
Source: PLoS One. 2026 Jul 31;21(7):e0351418. doi: 10.1371/journal.pone.0351418 (PMC13426950; doi:10.1371/journal.pone.0351418)
Supplement: S1 Table — Statistics for the highest-resolution shell are shown in parentheses. (DOCX) [file pone.0351418.s003.docx]

S1 Table. Data collection and refinement statistics for the HNA_closed_-KOD-H4 (PDB: 9R83) structure. Statistics for the highest-resolution shell are shown in parentheses.

|  | **HNA_closed_-KOD-H4** |
| --- | --- |
| **Data collection** | 09.12.2024 |
| Space group | P 2_1_ 2_1_ 2_1_ |
| a/b/c (Å) | 109.0/142.0/149.3 |
| α/β/γ (°) | 90.0 / 90.0 / 90.0 |
| Resolution range (Å) | 45.12 - 2.80 (2.83 - 2.80) |
| R-meas (%) | 19.1 (137.2) |
| CC1/2 (%) | 99.2 (62.5) |
| Mean I/sigma(I) | 5.72 (1.30) |
| Completeness (%) | 97.11 (98.7) |
| Redundancy | 4.8 (4.8) |
| **Refinement** | |
| Resolution (Å) | 45.12 - 2.80 (2.83 - 2.80) |
| Reflections used in refinement | 56216 (1854) |
| R_work_/R_free_ | 21.69 / 25.28 (33.96 / 36.96) |
| **Number of non-hydrogen atoms** | |
| macromolecules | 13182 |
| ligands | 322 |
| solvent | 73 |
| RMS (bonds) (Å) | 0.002 |
| RMS (angles) (°) | 0.50 |
| Ramachandran favored (%) | 94.84 |
| Ramachandran allowed (%) | 5.03 |
| Ramachandran outliers (%) | 0.13 |
| **B-factor** | |
| Average | 83.7 |
| macromolecules | 82.93 |
| ligands | 60.47 |
| solvent | 51.96 |
