# Supplementary material for: Crystal structure of a closed ternary complex of a HNA Reverse Transcriptase in complex with a HNA/DNA duplex
Source: PLoS One. 2026 Jul 31;21(7):e0351418. doi: 10.1371/journal.pone.0351418 (PMC13426950; doi:10.1371/journal.pone.0351418)
Supplement: S2 Table — (DOCX) [file pone.0351418.s004.docx]

S2 Table. Atomic B-factor comparison of the two polymerase complexes (A&B) within the asymmetric unit of HNA_closed_-KOD-H4 (PDB: 9R83).

| **Complex A** | **B-factor** | **Complex B** | **B-factor** |
| --- | --- | --- | --- |
| **Protein backbone** | 89.0 | **Protein backbone** | 75.0 |
| **Protein whole chain** | 89.6 | **Protein whole chain** | 76.2 |
| **p/t** | 102.3/97.3 | **p/t** | 94.6/93.4 |
| **HNA (total)** | 71.7 | **HNA (total)** | 70.0 |
| **HNA (t-t_-2_)** | 53.7 | **HNA (t-t_-2_)** | 54.7 |
| **dATP** | 51.1 | **dATP** | 50.3 |
