# Supplementary material for: Crystal structure of a closed ternary complex of a HNA Reverse Transcriptase in complex with a HNA/DNA duplex
Source: PLoS One. 2026 Jul 31;21(7):e0351418. doi: 10.1371/journal.pone.0351418 (PMC13426950; doi:10.1371/journal.pone.0351418)
Supplement: S3 Table — (DOCX) [file pone.0351418.s005.docx]

S3 Table. Local base parameters as given by 3DNA (49) for the active sites (t to t_-4_) within the duplexed structures of HNA_open_-KOD-H4, HNA_closed_-KOD-H4 and DNA_closed_-KOD-wt.

|  | **HNA_open_-KOD-H4** | Shear | Stretch | Stagger | Buckle | Propeller | Opening |
| --- | --- | --- | --- | --- | --- | --- | --- |
| **t** | **hC-nhdGTP** | 0.33 | -0.43 | -0.28 | **3.08** | **3.95** | -4.63 |
| **t_-1_** | **hT-dA** | 0.31 | -0.31 | 0.06 | **-0.76** | **-1.24** | -3.03 |
| **t_-2_** | **hG-dC** | 0.07 | -0.33 | 0.24 | **2.12** | **-3.13** | 1.79 |
| **t_-3_** | **hT-dA** | 0.22 | -0.22 | 0.47 | 5.74 | 1 | 1.87 |
|  | **HNA_closed_-KOD-H4** |  |  |  |  |  |  |
| **t** | **hT-dATP** | 0.11 | 0.07 | -0.27 | **1.29** | **2.73** | 14.68 |
| **t_-1_** | **hG-ddC** | 0.92 | -0.07 | -0.74 | **32.74** | **-17.11** | 1.34 |
| **t_-2_** | **hT-dA** | 0.05 | 0.00 | 0.25 | **19.62** | **0.78** | -3.07 |
| **t_-3_** | **dG-dC** | 0.33 | 0.23 | -0.57 | 17.35 | -16.38 | 8.95 |
|  | **DNA_closed_-KOD-wt** |  |  |  |  |  |  |
| **t** | **t_-+1_-t** | -0.01 | -0.24 | 0.06 | **-5.46** | **0.75** | 2.85 |
| **t_-1_** | **t-t_-1_** | -0.27 | -0.22 | 0.01 | **-8.37** | **-14.08** | 1.97 |
| **t_-2_** | **t_-1_-t_-2_** | -0.04 | -0.07 | -0.22 | **1.35** | **0.96** | -2.94 |
| **t_-3_** | **t_-2_-t_-3_** | -0.37 | -0.21 | -0.25 | -4.65 | -1.78 | 2.37 |
