# Supplementary material for: Crystal structure of a closed ternary complex of a HNA Reverse Transcriptase in complex with a HNA/DNA duplex
Source: PLoS One. 2026 Jul 31;21(7):e0351418. doi: 10.1371/journal.pone.0351418 (PMC13426950; doi:10.1371/journal.pone.0351418)
Supplement: S4 Table — HNA nucleotides are marked in magenta and distances were measured with Pymol [37]. (DOCX) [file pone.0351418.s006.docx]

S4 Table. Inter-phosphate distances of template strands at positions t to t_-3_. HNA nucleotides are marked in magenta and distances were measured with Pymol (37).

| **Complex A** | **HNA_open_-KOD-H4** | **HNA_Closed_-KOD-H4** | **DNA_closed_-KOD-wt** |
| --- | --- | --- | --- |
| **t-t_-1_** | 6.4 Å | 6.2 Å | 6.0 Å |
| **t_-1_-t_-2_** | 6.0 Å | 5.7 Å | 6.2 Å |
| **t_-2_-t_-3_** | 6.8 Å | 6.9 Å | 7.1 Å |
